# An Unusual Case of Hip Flexion Failure Caused by Bone Metastasis From Hepatocellular Carcinoma Infiltrating the Psoas Muscle

**DOI:** 10.7759/cureus.49996

**Published:** 2023-12-05

**Authors:** Yojiro Ishikawa, Satoshi Teramura, Kengo Ito, Takayuki Yamada

**Affiliations:** 1 Division of Radiology, Tohoku Medical and Pharmaceutical University, Sendai, JPN

**Keywords:** unable to walk, palliative radiation therapy, psoas major muscle, iliopsoas muscle, malignant psoas syndrome (mps)

## Abstract

When malignant tumors infiltrate the psoas muscle, they can result in what is referred to as malignant psoas syndrome (MPS). We are reporting this case as the malignant tumor had invaded the psoas muscle, and the clinical course of the patient differed from that of typical MPS. A 75-year-old male patient with liver cancer presented with pain around the right hip joint and difficulty in flexing the right hip joint, resulting in gait disturbance. There was no painful immobilization of the right hip, and the patient was able to extend the lower extremity. A CT scan revealed multiple liver tumors, multiple bone metastases, and swelling of the psoas muscle contiguous with the tumor at the lesser trochanter of the right femur. There were no apparent intracranial or spinal cord lesions, and no obvious abnormalities were detected around the psoas muscle in the abdominal cavity. Palliative radiation therapy was administered at a dose of 20 Gy in five fractions for pain relief. One month later, a follow-up CT scan presented no change in the shape of the tumor; however, the swelling of the right psoas muscle had improved. Unfortunately, the patient passed away 1 month after irradiation because of progressive liver and renal failure. When a patient with a malignant tumor presents with periprosthetic hip pain and hip flexion failure, one should consider the possibility of a malignant tumor in the lesser trochanter.

## Introduction

The condition of malignant tumor invasion into the psoas muscle is referred to as malignant psoas syndrome (MPS). MPS was first described in 1990 [1], and it is a rare condition caused by metastatic disease in the psoas muscle or direct invasion by a primary malignant tumor [2-10]. There have been few reports, and the mechanisms of the disease are not fully understood. MPS is known to be caused by the presence of imaging or pathologically proven malignant disease in the psoas muscle on the affected side, resulting in hip flexion retention (pain worsens with hip extension) and lumbosacral plexus disorders involving the first through fourth lumbar vertebrae. Physical examination often reveals neuropathy in the first to fourth lumbar vertebrae on the affected side and hip flexion fixation suggestive of psoas muscle spasm [2-4]. Furthermore, as these lesions progress, they may extend into the spinal canal, potentially causing epidural compression of the spinal cord or cauda equina, which can be a serious complication [3,11], sometimes necessitating bed rest, the use of a wheelchair for transfers, or a walker. The pathogenesis and therapeutic outcomes of MPS have been unclear; however, recent reports have shed light on the results of radiation therapy (RT), particularly in palliative care [3]. This case report describes a patient with multiple bone metastases from hepatocellular carcinoma invading the psoas muscle who, unlike typical MPS, presented with a rare clinical course of hip pain that did not worsen with hip extension, accompanied by weakness of the psoas muscle (hip flexion failure).

## Case presentation

**Figure 1:**
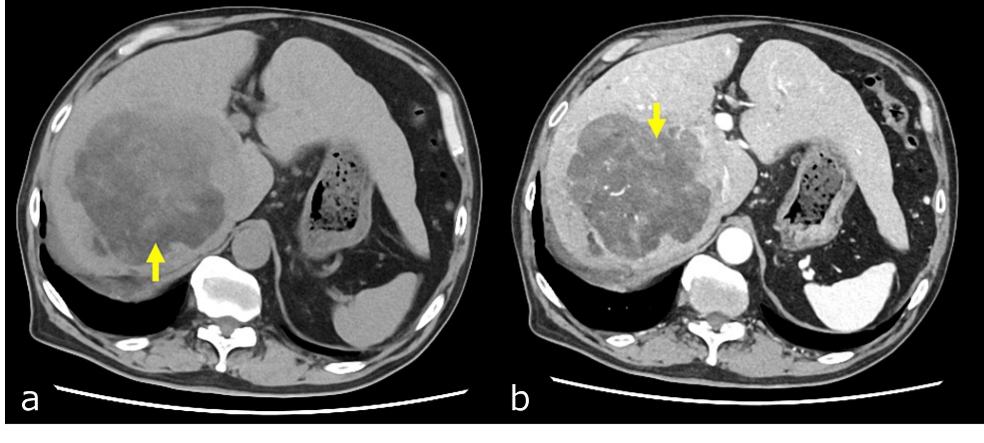
Abdominal CT scan Abdominal CT shows a 12 cm mass lesion in the posterior segment of the right hepatic lobe. The interior of the mass exhibits lower attenuation values than the liver parenchyma (a, indicated by yellow arrows). On contrast-enhanced CT, numerous blood vessels are observed internally, and early enhancement is clearly visible at the tumor margins (b, indicated by yellow arrows).

A 75-year-old man presented with abnormal liver function. He had a history of alcohol consumption and smoking but had quit smoking 20 years prior. Additionally, he had been diagnosed with diabetes mellitus, hyperlipidemia, hypertrophic cardiomyopathy, Adams-Stokes syndrome, pulmonary atresia, bronchial asthma, and prostate cancer. However, there were no active lesions associated with these conditions. No signs of liver disease were observed, and there was no history of previous liver disease treatment up to this point. Furthermore, there was no record of hepatitis virus infection up to this juncture. He did not exhibit any noticeable symptoms, and a thorough examination revealed no indications of muscle weakness, lower extremity paralysis, or neurological abnormalities. The patient's diagnosis included a 12 cm hepatocellular carcinoma (HCC) located in the right lobe of the liver, as confirmed by abdominal CT. Cirrhosis and ascites were also present (Figure 1).

**Figure 2:**
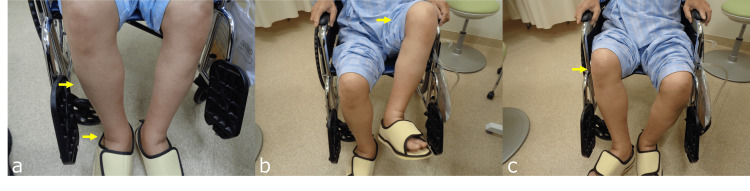
Physical examination findings Mild edema from the right thigh to the lower extremity (a, indicated by yellow arrows). The left lower extremity could be raised in a wheelchair sitting position (hip joint flexion) (b, indicated by yellow arrows). Raising the right lower extremity (hip joint flexion) was difficult (c, indicated by yellow arrows).

Laboratory investigations also revealed a total bilirubin (T-bil) level of 0.67 U/L (normal range: 0.4-1.5), an aspartate aminotransferase (AST) level of 66 U/L (normal range: 13-30), an alanine aminotransferase (ALT) level of 10 U/L (normal range: 10-42), a gamma-glutamyl transferase (γ-GTP) level of 269 U/L (normal range: 13-64), an albumin (Alb) level of 81.1 g/dL (normal range: 4.1-5.1), a prothrombin time (PT) level of 81.1% (normal range: 80-120), a PT-INR level of 1.14, an alpha-fetoprotein (AFP) level of 95.7 ng/mL (normal range: 0-10), and a protein induced by vitamin K absence or antagonist-II (PIVKA-II) level of 44.237 mAU/mL (normal range: 0-39). The child-pugh classification was determined to be Child A with two points attributed solely to ascites. Due to complications, resection surgery was considered difficult, and transarterial chemoembolization (TACE) was performed. No obvious neurological abnormalities were noted at this point, and the patient was able to walk. Three months later, the AFP level is 17.4 ng/mL, and the PIVKA-II level is 788 mAU/mL. Five months later, he underwent another TACE due to an increase in tumor markers (an AFP level of 40.9 ng/mL and a PIVKA-II level of 18,615 mAU/mL). During this time, the patient noticed that he had difficulty exerting strength in his right lower extremity. Manual muscle strength testing revealed a significant decrease in hip flexion strength to 1/5, while hip abduction, adduction, and extension remained within normal limits at 4/5. There were no signs of tendon hyperreflexia or other obvious neurological abnormalities. Cysto-rectal function remained normal. Both lower extremities showed mild edema. The patient experienced difficulty walking and relied on a wheelchair but was still able to stand. He also reported subjective symptoms of right hip pain, but there was no pain during flexion, and the pain did not worsen with other hip movements. When transferring to a wheelchair, the patient had to perform a specific action of lifting his right leg with his hand because he was unable to flex his right hip joint (Figure 2).

**Figure 3:**
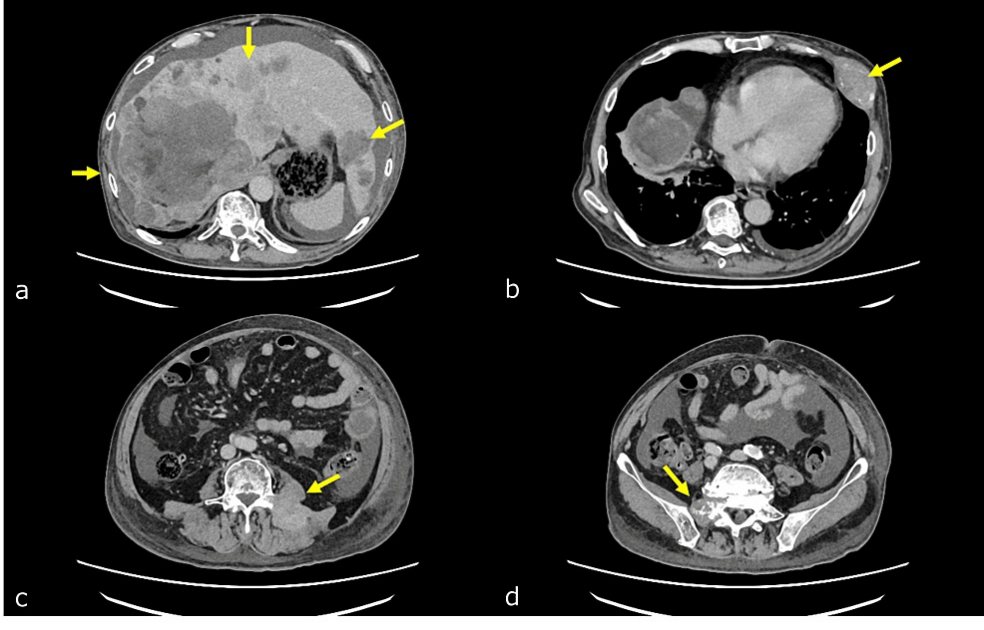
CT scan of the trunk A CT scan of the trunk, lumbar region, and pelvis revealed advanced liver metastases (a, indicated by yellow arrow) and metastatic tumors in the rib (b, indicated by yellow arrow), sacrum (c, indicated by yellow arrow), and sacrum (d, indicated by yellow arrow) (Figure 3).

**Figure 4:**
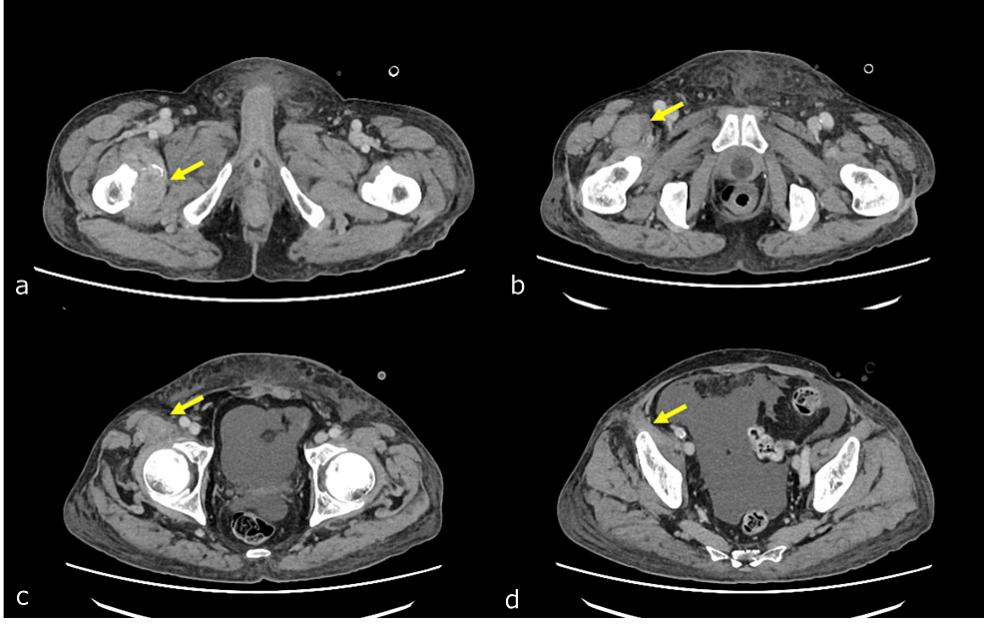
Pelvic CT scan Bone metastasis was observed in the right femoral lesser trochanter (a, indicated by the yellow arrow), along with infiltration and muscle thickening near the right iliopsoas muscle transversion (b-c, indicated by the yellow arrow), and muscle thickening extending to the right psoas muscle near the iliac crest (d, indicated by the yellow arrow).

A CT scan of the trunk, lumbar region, and pelvis revealed advanced liver metastases and metastatic tumors in the lumbar spine, sacrum, and right femoral lesser trochanter (Figure 3). The right psoas muscle showed consecutive enlargement due to the metastasis of the right femoral lesser trochanter (Figure 4). A lumbar MRI scan showed no obvious spinal cord involvement, and a head MRI scan revealed no brain metastases.

**Figure 5:**
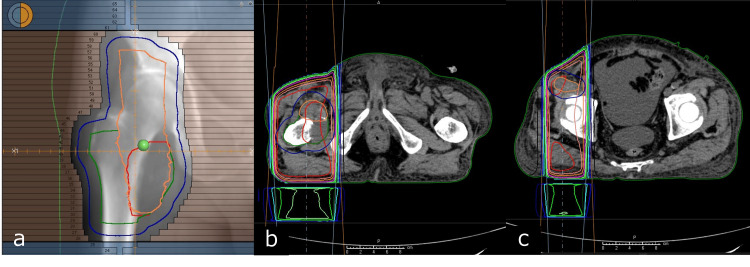
Radiation therapy field and dose distribution RT plan was generated in the Raystation treatment planning system (RaySearch Laboratories AB, Stockholm, Sweden). The radiation therapy fields are displayed in the beam's eye view (a), as well as in the axial images (b,c). The gross tumor volume was defined as the bone metastasis in the popliteal region. The clinical target volume (CTV) was delineated to encompass the area surrounding the femur, including the tumor and the thickened iliopsoas muscle. The planning target volume (PTV) was subsequently defined as the CTV plus an additional 1.0 cm margin. Ten-megavolt (10 MV) X-rays were administered from two anterior and posterior quadrants.

Neurological examination revealed weakness limited to hip flexion with no evidence of upper motor neuron involvement, leading to suspicion of peripheral nerve palsy or myogenic palsy. The patient was diagnosed with hip flexion impairment likely resulting from right psoas muscle weakness or a fracture of the right femoral lesser trochanter. Due to the progression of liver metastases and multiple bone metastases, the patient received palliative treatment and underwent RT for the right femoral metastasis. Palliative RT of 20 Gy in five fractions was administered for metastasis of the right femoral lesser trochanter (Figure 5).

**Figure 6:**
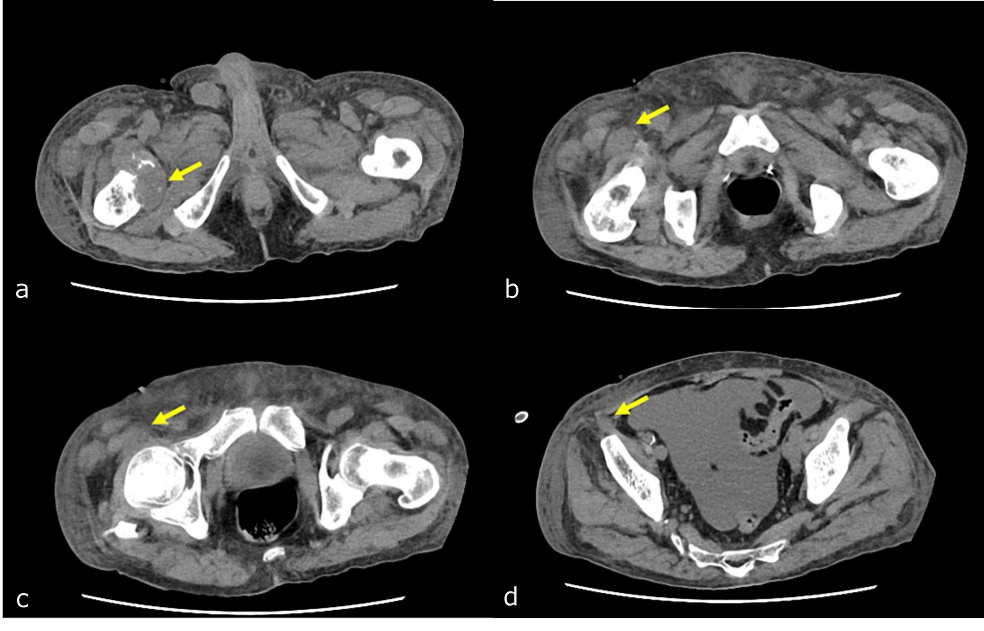
CT scan of the pelvic region one month after radiation therapy The size of the bone metastasis in the right femoral lesser trochanter remained similar before radiation therapy (a, indicated by the yellow arrow). However, there was an improvement in the infiltration and muscle thickening near the right iliopsoas muscle (b-d, indicated by the yellow arrow).

The pain level before the start of RT was rated at 5-7 points on a numerical rating scale (NRS). He was taking non-steroidal anti-inflammatory drugs (NSAIDs) on an ad-hoc basis. Pain improved after approximately one week of RT, and the patient had an NRS score of 0-1. However, muscle weakness in the right hip flexion did not completely improve even after one month of RT. Due to the deterioration of his condition, he was unable to undergo sensory diagnoses, but he mentioned that he was able to flex his hip joints slightly. He was scheduled to be admitted to the palliative care ward, but within one month of RT, renal and hepatic failure progressed, leading to his passing in the emergency room (ER). A review of the CT scan obtained in the ER showed no change in the size of the mass in the right femoral lesser trochanter area, but the thickening of the right psoas muscle had improved (Figure 6).

## Discussion

This case report described a patient with metastatic HCC located in the lesser trochanter of the femur, invading the area around the psoas muscle. While bone metastasis from liver cancer is less common compared to lung, breast, and prostate cancers, the incidence of bone metastasis in patients with hepatocellular carcinoma has been reported to range from 1.97 to 23.59% [12]. Bone metastases in the lower leg are also reported to be infrequent (5.0%). In this case, bone metastasis of HCC occurred in the lesser trochanter of the femur, which is relatively uncommon as a site for bone metastasis in terms of frequency [13].

A condition in which a malignant tumor invades the psoas muscle is often encountered in daily medical practice. One well-known example is referred to as MPS [2-10]. Since its first description in 1990 [1], its pathogenesis and therapeutic outcomes have not been fully understood. Regarding the nerves and mechanisms related to the development of pain, malignant lesions of the psoas major and proximal lumbosacral plexus and its branches can cause pain, with associated pain patterns usually seen in the groin, thighs, and anterior abdominal wall. Possible causes include neuropathic pain because of lumbosacral plexopathy, somatic nociceptive pain, and muscle spasms because of psoas muscle involvement [2,3,14]. The psoas, inguinal, and femoral inguinal nerves descend to the surface of the muscle posterior to the iliopsoas fascia [15,16]. However, in this case, the pain was caused by a tumor in the trochanteric femoral region and did not affect the psoas major, inguinal, or femoral inguinal nerves, resulting in no painful fixation of the hip joint. There have been no previous reports of clinical findings where there is no painful fixation of the hip joint despite the involvement of the iliopsoas muscle and limited hip flexion. Although similar to MPS in clinical findings related to the hip joint, we believe that cases like this one need to be distinguished from typical MPS. Another characteristic finding in this case was that although the patient had apparent difficulty walking and used a wheelchair, there was no paralysis of the lower leg muscles. Only hip flexion was reduced, which forced the patient to rely on a wheelchair. The psoas muscle also plays a role in connecting the pelvis to the lower extremities, and its connection to the femur plays an important role in this case [17,18]. While paralysis has been reported in MPS [3], it is typically because of special circumstances such as spinal cord lesions and differs from the pathophysiology in this case [19,20]. In daily clinical practice, when only one side of the iliopsoas muscle is weakened, as in this case, it is crucial to consider the possibility of a lesion in the lesser trochanter. Conversely, in cases where a patient arrives at the hospital in a wheelchair, as in the present case, without a thorough examination, there is a likelihood that the physician may not recognize that the cause of their gait disturbance is solely because of weakness in the iliopsoas muscle and hip flexion.

In a prospective Phase II study involving 60 patients with gastrointestinal cancer (including HCC, n=25) and bone metastases, a combination of zoledronic acid was reported to achieve pain response rates of 95% and 96% [21]. Treatment options for MPS include analgesics and opioids, although radiation therapy is often used [2,22]. Previous studies have demonstrated the efficacy of radiation therapy for MPS [23,24]. These prior studies on radiation therapy for MPS involved only a limited number of cases, and, to our knowledge, there is only one report, a single-center retrospective study, on treatment outcomes. This study examined radiation therapy for MPS symptoms in 22 patients and reported that 15 patients (68%) experienced symptom relief or decreased analgesic use without serious adverse events [3]. In this case, patients also reported an improvement in pain. A posttreatment CT scan showed that the tumor had shrunk, and the iliopsoas muscle had increased in size. However, the patient was still unable to walk, in part because of the progression of the lesion. It is important to note that this case responded relatively well to radiation therapy. Typically, positioning MPS patients in the supine position during radiotherapy is challenging because of hip flexion fixation. It has been reported that radiotherapy is administered after pain management, such as induction of irradiation or epidural anesthesia, following adequate opioid administration [14].

## Conclusions

In patients with malignancy, suspicion should arise regarding a neoplastic lesion in the lesser trochanter of the femur, keeping MPS in consideration, particularly when presenting with (i) hip pain on one side, (ii) the absence of painful hip fixation, and (iii) gait disturbances resulting from hip flexion failure. The present case will provide valuable insights for oncologists encountering similar conditions. In this particular case, radiotherapy successfully relieved pain, although the restoration of leg muscle strength remained challenging. Nevertheless, with a precise diagnosis and prompt initiation of treatment, it may be possible to restore leg muscle strength.
